# Antagonistic Efficacy of Symbiotic Bacterium *Xenorhabdus* sp. SCG against *Meloidogyne* spp.

**DOI:** 10.4014/jmb.2404.04003

**Published:** 2024-07-15

**Authors:** Jong-Hoon Kim, Byeong-Min Lee, Hyung Chul Lee, In-Soo Choi, Kyung-Bon Koo, Kwang-Hee Son

**Affiliations:** 1 Microbiome Convergence Research Center, Korea Research Institute of Bioscience and Biotechnology, Daejeon 34141, Republic of Korea; 2 Department of Biotechnology, Pukyong National University, Busan 48513, Republic of Korea; 3 ECOWIN Co., Ltd., Daegu 42993, Republic of Korea; 4 Nematode Research Center, Life and Industry Convergence Research Institute, Pusan National University, Miryang 50463, Republic of Korea

**Keywords:** Entomopathogenic nematode, *Xenorhabdus*, nematicidal activity, root-knot nematode, biological control

## Abstract

The inhabitation and parasitism of root-knot nematodes (RKNs) can be difficult to control, as its symptoms can be easily confused with other plant diseases; hence, identifying and controlling the occurrence of RKNs in plants remains an ongoing challenge. Moreover, there are only a few biological agents for controlling these harmful nematodes. In this study, *Xenorhabdus* sp. SCG isolated from entomopathogenic nematodes of genus *Steinernema* was evaluated for nematicidal effects under in vitro and greenhouse conditions. The cell-free filtrates of strain SCG showed nematicidal activity against *Meloidogyne* species J2s, with mortalities of > 88% at a final concentration of 10%, as well as significant nematicidal activity against the three other genera of plant-parasitic nematodes in a dose-dependent manner. Thymine was isolated as active compounds by assay-guided fractionation and showed high nematicidal activity against *M. incognita*. Greenhouse experiments suggested that cell-free filtrates of strain SCG efficiently controlled the nematode population in *M. incognita*-infested tomatoes (*Solanum lycopersicum* L., cv. Rutgers). In addition, a significant increase in host plant growth was observed after 45 days of treatment. To our knowledge, this is the first to demonstrate the nematicidal activity spectrum of isolated *Xenorhabdus* species and their application to *S. lycopersicum* L., cv. Rutgers under greenhouse conditions. *Xenorhabdus* sp. SCG could be a promising biological nematicidal agent with plant growth-enhancing properties.

## Introduction

Approximately 4,000 species of plant-parasitic nematodes (PPNs), some of which cause significant damage to industries such as agriculture and forestry, are distributed worldwide [1, 2]. These nematodes account for up to approximately 25% of annual losses in agriculture, resulting in economic damage of approximately $157 billion [3, 4]. Based on their feeding mechanisms, PPNs are classified into migratory ectoparasites, migratory endoparasites, and sedentary endoparasites. Among these, root-knot nematodes (RKNs), a major group of sedentary endoparasites, cause the greatest damage to crops [5]. There are 98 known genera of RKNs, among which the *Meloidogyne* species are known to cause the greatest damage [6]. Infective second-stage juveniles (J2s) of these RKNs physically damage plant roots via stylets, such as hollow mouth spears, and penetrate plant roots using cell wall-degrading enzymes. This induces specific root cells to expand and become multinucleated giant cells, thereby depriving host plants of nutrients [7].

There are various methods for controlling RKNs, including synthetic nematicides, botanical essential oils, biological control, and resistant cultivars, for use alone or in combination for better control [8]. The most commonly used synthetic nematicides are chemical fumigants (*e.g.*, as methyl bromide, dichloropropane, and dimethyl disulfide) and synthetic compounds (*e.g.*, as fosthiazate, oxamyl, and fluensulfone) [9]. However, chemical control is limited or prohibited because of several factors including the occurrence of multidrug resistance in RKNs, environmental contamination, persistence, and biotoxicity [10]. This necessitates substitutes to reduce the use of chemical pesticides for sustainable pest control. Biological control methods using microorganisms such as fungi and bacteria offer a promising alternative to chemical control methods for RKNs [11]. Fungi, such as *Paecilomyces*, *Trichoderma*, and *Aspergillus* effectively control RKNs in various crops [12-15]. Plant growth-promoting rhizobacteria also have a high control potential against RKNs in various crops. Certain bacterial species, including *Bacillus cereus*, *Bacillus subtilis*, *Pseudomonas putida*, *Pseudomonas fluorescens*, and *Serratia proteamaculans* have shown high potential for controlling RKNs [16]. *B. altitudinis* KMS-6 increased plant growth and yield in eggplants and cucumbers and reduced damage to *M. javanica* [17]. Similarly, *P. fluorescens* CHA0, which produces hydrogen cyanide, greatly reduces egg hatching and exhibits high nematicidal activity against *M. javanica* [18]. These microorganisms can be used alone or in combination with other control methods for more optimized control of RKNs while reducing the use of chemical pesticides for sustainable agriculture.

*Xenorhabdus* is an entomopathogenic bacterium that forms a natural mutualistic relationship with the entomopathogenic nematodes (EPNs) of the genera *Steinernema* and *Heterorhabditis* [19]. The EPNs-*Xenorhabdus* complexes release the symbiotic bacterium *Xenorhabdus* after entering the host, which produces various metabolites that kill the host and protect the cadaver from saprophytic microbial competitors and animal scavengers [20-22]. Furthermore, recent studies have suggested that the *Xenorhabdus* species could be a novel resource for managing RKNs using nematicidal secondary metabolites [23-25]. However, nematicidal activity of *Xenorhabdus* against *Meloidogyne* spp. and their green-house applications have not comprehensively studied. Therefore, this study aimed to screen, identify, and evaluate nematicidal *Xenorhabdus* species isolated from EPN against *Meloidogyne* species under laboratory conditions. We also investigated the effects of fermentation supernatant of isolated *Xenorhabdus* on potted tomatoes (*Solanum lycopersicum* L. cv. Rutgers) in soils infested with *M. incognita* under greenhouse conditions.

## Materials and Methods

### Entomopathogenic Nematodes

EPNs were isolated from sandy soil samples collected from forest sites in Pocheon-si, Gyeonggi-do, Republic of Korea using the *Galleria mellonella*-baiting method [26]. *Galleria mellonella* (Lepidoptera: Pyralidae) larvae were reared on artificial diet at 25°C in a dark chamber [27]. After 5 days of baiting, the dead *G. mellonella* larvae showing typical morphological symptoms of EPN infection were subsequently transferred to white traps [28]. The third-stage infective juveniles (IJ) were harvested in the subsequent days and stored at 10°C.

To identify species of isolated EPNs, genomic DNA of each isolate was extracted using a standard phenolchloroform extraction method and a partial 28S rDNA gene was amplified by polymerase chain reaction (PCR) using primers 539_F (5'-GGATTTCCTTAGTAACTGCGAGTG-3') and 535_R (5'-TAGTCTTCGCCCCTATACCCTT- 3'). Purified products were sequenced by Macrogen, Inc., (Republic of Korea). The sequence of the 28S rDNA gene was compared with that of the type strains available at the National Center for Biotechnology Information (http://www.ncbi.nlm.nih.gov/GenBank/index.html) to identify closely related species. The molecular phylogeny of 28S rDNA was inferred using the neighbor-joining method in MEGA X software [29].

To determine the insecticidal activity of the seven isolated EPNs, bioassays were performed against 3rd instar larvae of *G. mellonella*. Basal sections of filter paper (Advantec 2, Toyo Roshi Kaisha, Ltd., Japan) were placed in 90 mm petri dishes (SPL Life Sciences, Republic of Korea). Thirty larvae, individually inoculated with 100 IJs of seven isolated EPNs, were placed in each Petri dish. Distilled water was used as a negative control. The treated larvae were maintained under rearing conditions, and larval mortality was scored at 24 h intervals for 5 days. All assays were repeated three times under the same conditions.

### Isolation and Identification of Symbiotic Bacteria

The last instar larvae of *G. mellonella* infected with IJs of *Steinenerma* sp. 13-0112 were immersed in 70% (v/v) ethanol for 1–2 min to remove the surface contaminants and then washed twice with sterile distilled water. Then, hemolymph samples were obtained with a sterilized insulin syringe (BD Ultra-Fine Insulin Syringe, USA) and spread onto nutrient bromothymol blue-triphenyltetrazolium chloride agar (0.025 g bromothymol blue, 0.004 g triphenyl tetrazolium chloride, 37 g nutrient agar, 1 L distilled water) to isolate the *Xenorhabdus* species [30]. Following incubation at 28°C for 2 days, a single bacterial blue colony was selected for further experiments. The bacterial strain was isolated and stored at −70°C in R2A broth containing 25% sterilized glycerol.

Genomic DNA was extracted using a standard phenol-chloroform extraction method, and a partial 16S rRNA gene was amplified by PCR for molecular identification of the bacterial isolates. Universal primers 27F (5'-AGAGTTTGATCMTGGCTCA-3') and 1492R (5'-TACGGYTACCTTGTTACGACTT-3') were used. Purified products were sequenced by Macrogen, Inc. The 16S rRNA gene sequence was compared with that of the type strains available in the EzBioCloud database (ChunLab Inc., Republic of Korea) to identify closely related species. The molecular phylogeny of 16S rRNA was inferred using the neighbor-joining method in the MEGA X software.

### Nematicidal Activity

The *Meloidogyne incognita* used in this study was originally collected from the oriental melon (*Cucumis melo* L. var. makuwa) roots in a commercial greenhouse at Yesan-ri (Republic of Korea) and then maintained on tomatoes (*Solanum lycopersicum* L., cv. Rutgers, Seedway, USA) under greenhouse conditions at 28 ± 2°C [31].

The egg masses were obtained from infected tomato using a 0.5% NaOCl solution according to the methods reported by Hussey and Barker with minor modifications, and incubated at 28°C for 24 h in distilled water using a modified Baermann funnel to obtain second-stage juveniles (J2s) of *M. incognita* [32]. The hatched J2s were used for in vitro and in vivo experiments. The other pure cultured-nematodes (*M. javanica*, *M. hapla*, *M. arenaria*, *Ditylenchus destructor*, *Aphelenchoides subtenuis*, and *Heterodera trifolii*) for the activity spectrum analysis were provided from the Nematode Research Center, Life and Industry Convergence Research Institute, Pusan National University (Republic of Korea).

Bacterial isolates were fermented in a 500 ml baffled Erlenmeyer flask containing 100 ml of LB broth (BD Difco, USA) which was incubated on a shaking incubator (200 ×*g*) at 28°C for 48 h. After fermentation, supernatants were separated by centrifugation at 10,000 rpm for 15 min at 4°C and subsequently filtered using a 0.22 μm pore filter (Millipore, USA). An aliquot containing 50 fresh hatched-J2s in 90 μl sterilized water was transferred to the each well of a 96-well plate (SPL Life Sciences Co. Ltd., Republic of Korea) and treated with the 10 μl of cell-free filtrates at a final concentration of 10% (v/v). LB broth was used as the negative control, and 2,000-fold diluted Sunchungtan 150EC (150 μg/ml of fosthiazate, Farm Hannong Co., Republic of Korea) as the positive controls. The 96-well plates were incubated at 28°C for 48 h. After incubation, the survival of J2s in each treatment was observed using a stereomicroscope (Olympus SZ61, Olympus Corp., Japan); J2s were declared dead when they appeared straight and immobile after stimulation with a fine needle. The relative mortality rate was calculated using the following formula: [(mortality rate of treatment-mortality rate of negative control)/(1-mortality rate of negative control)], according to Abott’s formula [33]. All experiments were performed in triplicate wells and repeated three times under the same conditions.

### Isolation and Identification of Nematicidal Compound from Strain SCG Cultures

Strain cultures were prepared as described above and centrifuged at 10,000 rpm for 10 min. The collected supernatant was then sequentially extracted with an equivalent volume of *n*-hexane, diethyl ether, and ethyl acetate. Different solvent layers were concentrated in a rotary vacuum to obtain the dry extracts and redissolved in dimethyl sulfoxide (DMSO) for activity tests. The nematicidal activity of the extracts was determined as described above. An aliquot containing 50 fresh hatched-J2s in 98 μl sterilized water was transferred to the each well of a 96-well plate and treated with the 2 μl of the solvent extract. The final concentrations of solvent extracts were 0.1, 0.25, 0.5, and 1.0 mg/ml, and 2 μl of DMSO was used as a control. The concentrated ethyl acetate extract, which showed the highest nematicidal activity, was separated using a Biotage Isolera (USA) automated purification system equipped with a UV detector at 254 nm and a SNAP column cartridge (100 g silica gel). Separation was carried out with a stepwise chloroform/methanol gradient of increasing polarity (50:1, 20:1, 10:1, 5:1, 4:1, and 1:1). One of the fractions that showed high nematicidal activity was separated by preparative reverse-phase HPLC (solvent: methanol/water 10:90 v/v, flow rate: 1.0 ml/min) using an Inno C18 column (5 μm, 250 × 4.6 mm; Young Jin Biochrom, Republic of Korea) and further purified using a U-VDSpher PUR 100 C18E column (1.8 μm, 50 × 2.0 mm VDS Optilab, Germany) with acetonitrile/water (70:30) containing 0.2% formic acid at a flow rate of 0.3 ml/min. Electrospray ionization-mass spectrometry (ESI-MS) was performed using a Q-TOF 5600 (AB Sciex, Canada) high-resolution liquid chromatography tandem mass (LC/MS/MS) spectrometer. The ^1^H and ^13^C nuclear magnetic resonance (NMR) (600 MHz) spectra were obtained using a high-resolution Avance 600 NMR spectrometer (Bruker, Germany) with methanol as the solvent. The chemical structure of each compound was determined by comparing the NMR and MS data with published literature values. The nematicidal activity of the purified compound was determined as described above treated with the final concentrations of compound were 0.05, 0.1, and 0.25 mg/ml, and 2 μl of DMSO was used as a control.

### Pot Experiments under Greenhouse Condition

An experiment was performed in the controllable greenhouse of the Nematode Research Center, Life and Industry Convergence Research Institute, Pusan National University (Republic of Korea) with a temperature range of 25 ± 3°C and 70% relative humidity, and 12 h light/12 h dark cycle. Pots (12 cm in diameter and 10 cm in depth) were filled with 500g of soil from a commercial greenhouse in Seongju-gun, Republic of Korea (35°55'32.2"N 128°17'13.8"E) and inoculated with *M. incognita* (1 J2/g of soil) in 1 mL of sterilized water. Cell-free filtrates of the bacterial isolates were prepared as described above. After 24 h of inoculation, an experiment was carried out with three treatments: (1) cell-free filtrates (10%, 5%, and 1%), (2) 2,000-fold diluted Sunchungtan 150EC (150 μg/mL of fosthiazate, Farm Hannong Co.) as positive control, and (3) LB broth as negative control. One week after the treatment, tomato seedlings (*Solanum lycopersicum* L. cv. Rutgers, USA) at the two leaf stage were transplanted into pots (one for each pot). Plant growth parameters (fresh height and weight of shoots and roots) and nematode parameters (number of egg masses on each tomato root and population of nematodes in the soil of each replicate pot) were determined 45 days after transplanting. The number of egg masses was determined using phloxine B staining. The population of nematodes in 500 g of soil from each replicate pot was determined according to Coolen’s method under a stereomicroscope (Olympus SZ61) [34]. All experiments were performed in triplicate.

### Statistical Analysis

One-way ANOVA was performed using SPSS software (version 24; SPSS, Inc., USA). The mean values were compared using Scheffé’s method, and *p* values < 0.05 were considered statistically significant.

## Results

### Insecticidal Activities of Isolated Entomopathogenic Nematodes

In total, seven EPN were isolated from sandy soils of forest sites (Republic of Korea). Based on the 28S rDNA sequence, all isolated EPNs belonged to the *Steinernema* species (Fig. S1A). To evaluate the virulence of the 7 isolated EPNs, 3rd instar larvae of *G. mellonella* were treated with 100 IJs. All seven isolated EPNs showed high levels of larvicidal activity with mortalities of > 60% (Fig. S1B). Among the seven isolates, 21-0118 showed the highest level of virulence, with mortalities of 100%. Additionally, the average lifespan of the infected larvae was inversely proportional to larval mortality, and the lifespan was lower at higher mortality rates. *Steinenerma* sp. 21-0118 exhibited the highest virulence was selected for further studies.

### Isolation and Identification of Nematicidal *Xenorhabdus* Species

**Fig. 1:**
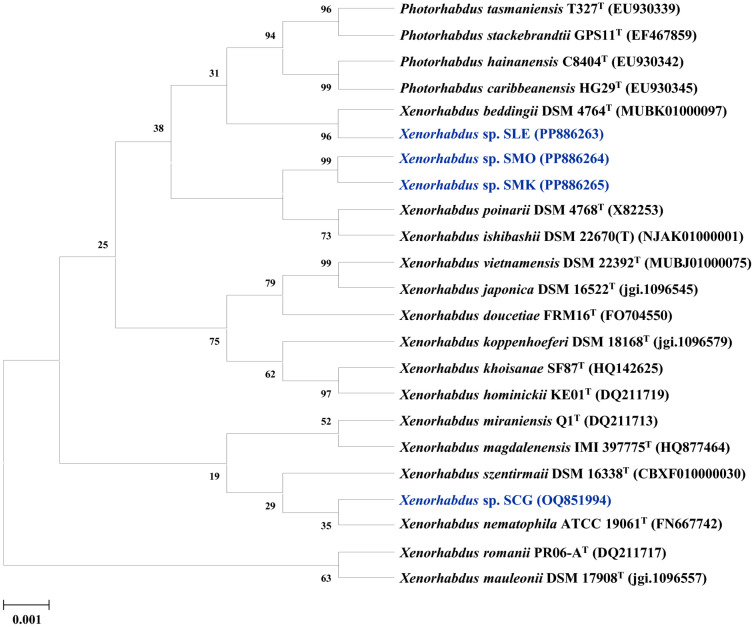
Phylogenetic relationship of the isolated *Xenorhabdus* strains based on 16S rRNA gene sequence. Neighbor-joining phylogenetic tree based on 16S rRNA gene sequences and closely related species constructed using MEGA X software. Numbers at each branch indicate the bootstrap percentage of 1,000 replications.

On the basis of blue colony morphology, we isolated four strains from *Steinenerma* sp. 21-0118. For phylogenetic profiling, the partial 16S rRNA gene sequences of the four isolates were compared with that of the type strains available in the EzBioCloud database. The results showed that these four isolates belonged to *Xenorhabdus* species (Table S1). A phylogenetic tree constructed using the neighbor-joining method showed that the isolate SCG was most closely related to *Xenorhabdus nematophila* ATCC 19061 (GenBank accession number FN667742), with 98.41% 16S rRNA nucleotide sequence similarity (Fig. 1). The isolates SMO and SMK were most closely related to *Xenorhabdus bovienii* subsp. bovienii (X82252) with 99.86% and 99.58% similarity, respectively. On the contrary, isolate SLE showed a 16S rRNA similarity of 99.80% to *Xenorhabdus beddingii* (MUBK01000097). The 16S rRNA nucleotide sequence was deposited in GenBank under accession number PP886264 (strain SMO), OQ851994 (strain SCG), PP886263 (strain SLE), and PP886265 (strain SMK).

**Fig. 2:**
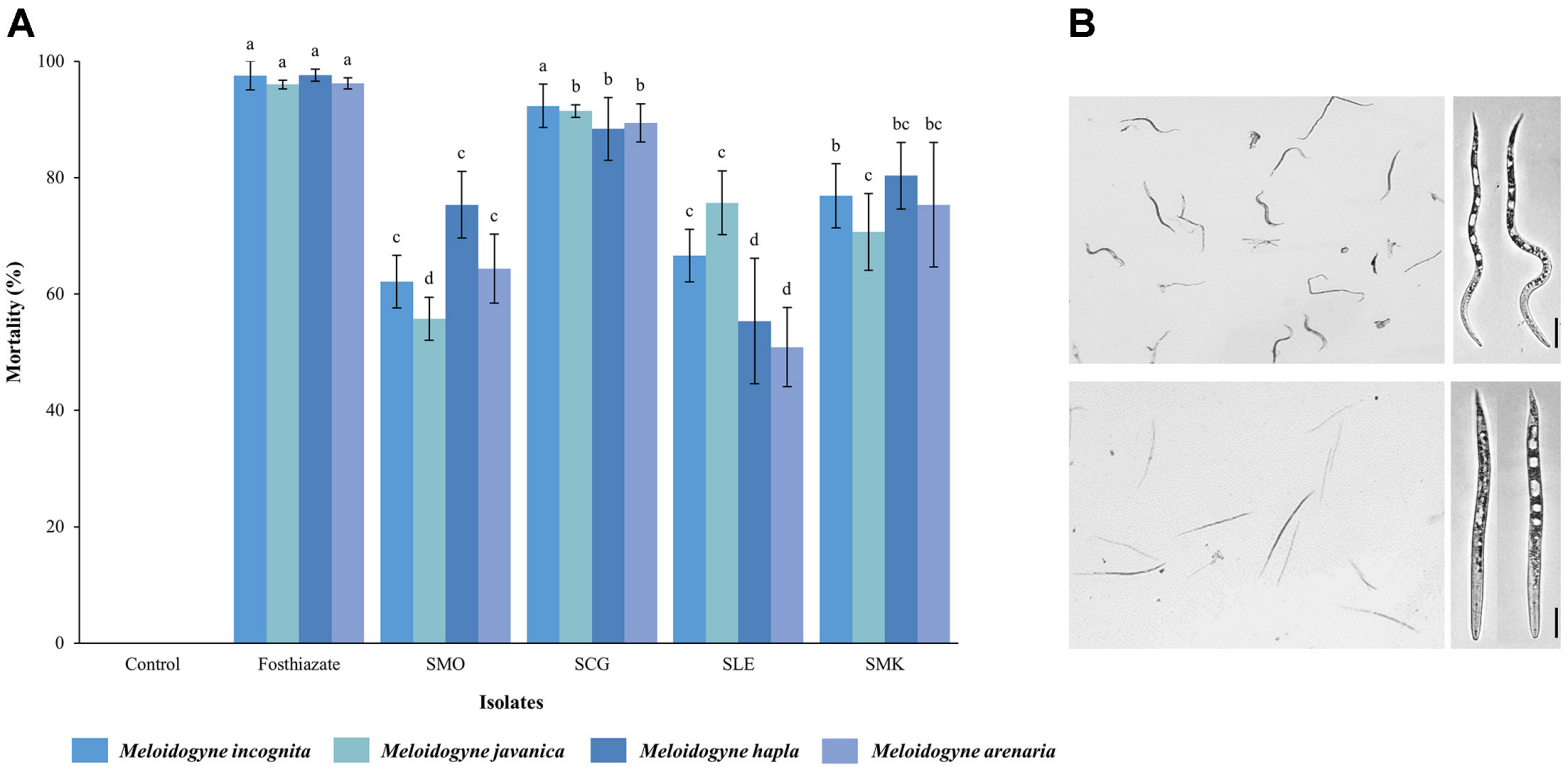
Nematicidal activities of isolated bacteria against the J2s of four *Meloidogyne* species. (**A**) Mortality rate of the J2s of Meloidogyne after 48 h treated with 10% cell-free filtrates of isolated bacteria. Sunchungtan 150EC (150 μg/ml of fosthiazate) were used as the positive control, while LB broth was used as the negative control. (**B**) Morphological observation of the assessed nematodes with active nematodes (upper) and the dead nematodes with straight form and immobility (below) post-stimulation using a fine needle. All experiments were performed in triplicate wells and repeated three times under the same conditions. Different letters above the error bars indicate significant differences by Scheffé's test (*p* < 0.05). Scale bar: 20 μm.

Four isolated strains were evaluated for nematicidal activity against four *Meloidogyne* species J2s. Among the isolated strains, the cell-free filtrates of strain SCG showed the highest nematicidal activity against all of four *Meloidogyne* species J2s, with mortalities exceeding 88% (*M. incognita*, 92.36%; *M. javanica*, 91.46%; *M. hapla*, 88.41%; *M. arenaria*, 89.43%) at a final concentration of 10% (Fig. 2A). The mortality rate of J2s was > 96% when treated with the positive control fosthiazate (150 μg/ml). All the dead nematodes exhibited a typical antagonistic straight posture with no vitality when stimulated using a fine needle (Fig. 2B). Strain SCG exhibited the highest nematicidal activity against the J2s of *Meloidogyne* species and was selected for further studies.

### Nematicidal Spectrum of SCG Strain

**Fig. 3:**
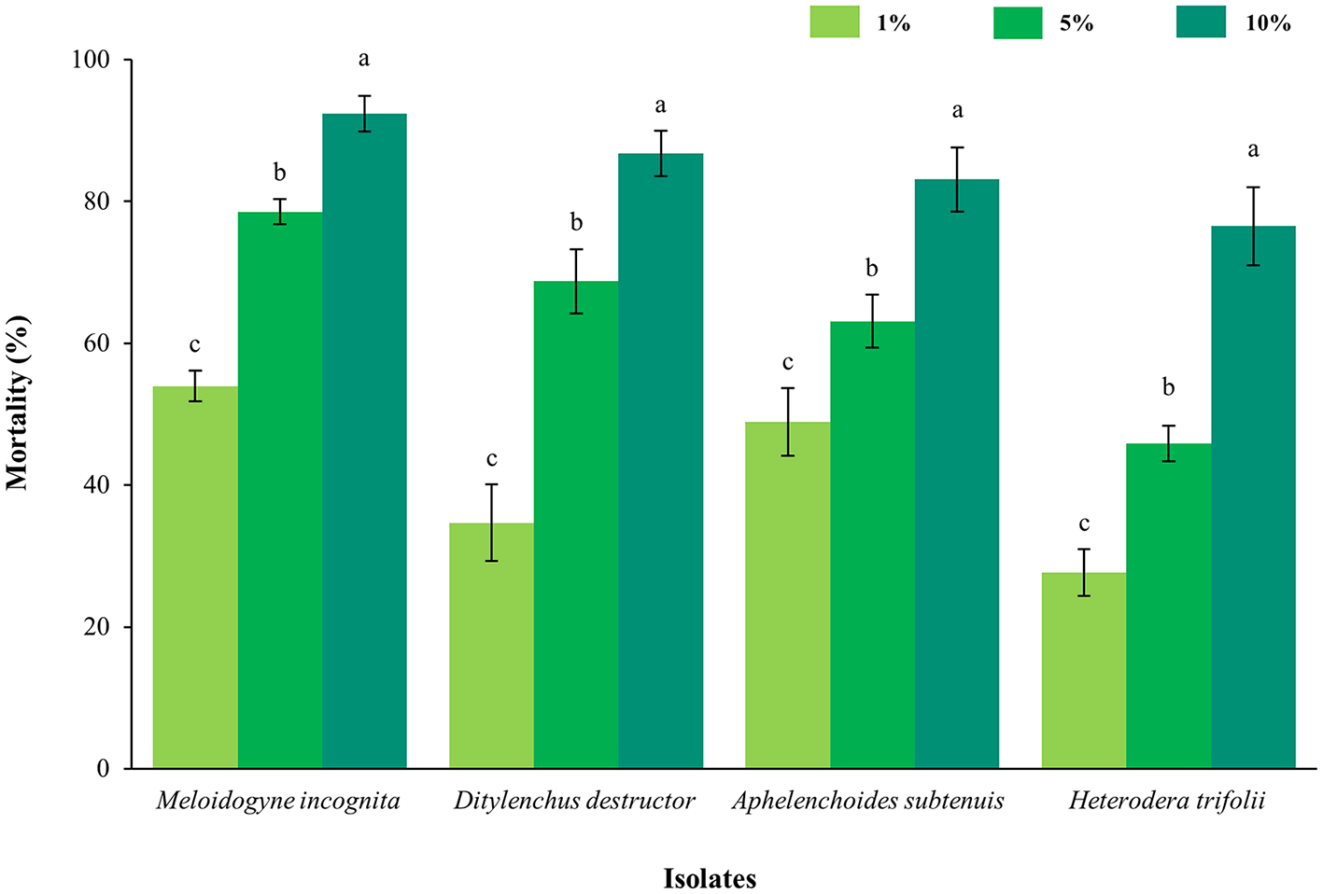
Assessments of the nematicidal spectrum of cell-free filtrates of SCG strain against the second-stage juveniles of four plant parasitic nematodes. Sunchungtan 150EC (150 μg/ml of fos-thiazate) were used as the positive control, while LB broth was used as the negative control. All experiments were performed in triplicate wells and repeated three times under the same conditions. Different letters above the error bars indicate significant differences by Scheffé's test (*p* < 0.05).

To determine the nematicidal potential of strain SCG, the nematicidal activity spectrum was assessed against J2s of four plant-parasitic nematodes (*M. incognita*, *Ditylenchus destructor*, *Aphelenchoides subtenuis*, and *Heterodera trifolii*) with different concentrations of cell-free filtrates (Fig. 3). Overall, the mortality rate of J2 increased proportionally to the concentration of bacterial cell-free filtrates. Assessment of the activity spectrum determined in bioassays showed remarkable broad-spectrum activity with percentage mortality rates of 92.36%, 86.78%, 83.12%, and 76.54%. In addition, *M. incognita* was the nematode most susceptible to cell-free filtrates of the SCG strain at all concentrations.

### Purification of Nematicidal Compound from SCG Strain

**Fig. 4:**
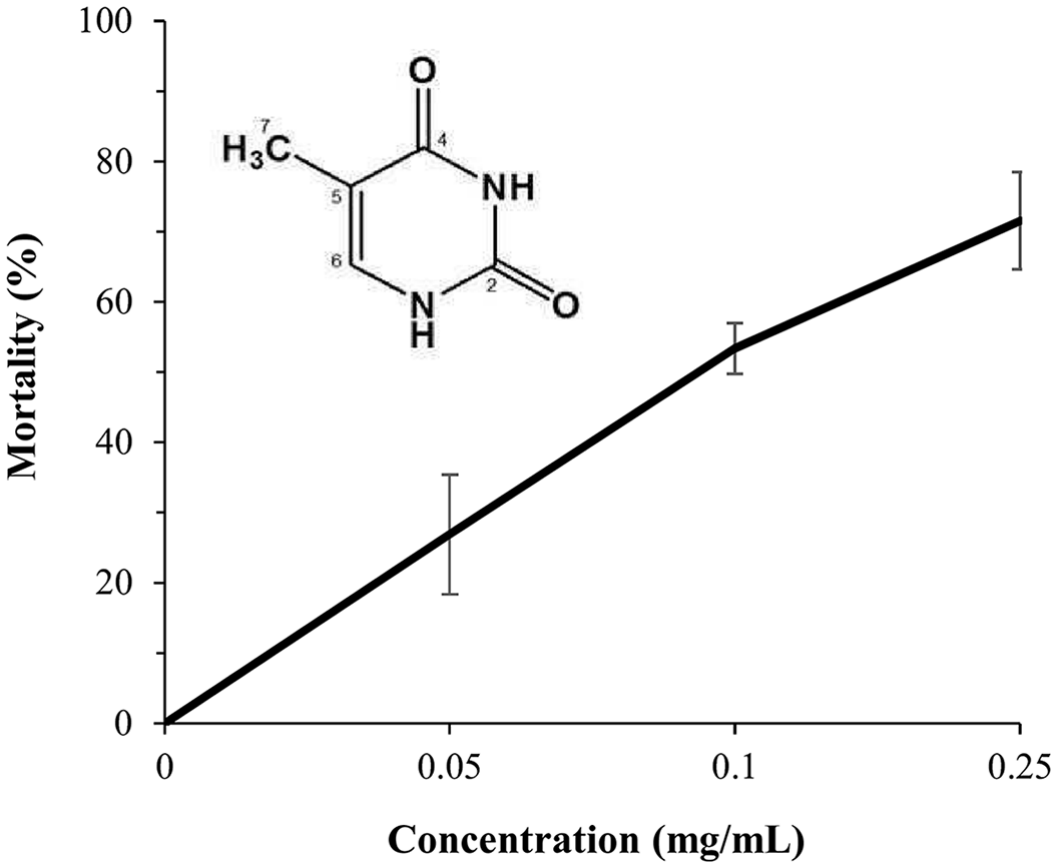
Concentration-dependent nematicidal activity of thymine from strain SCG. All experiments were performed in triplicate wells and repeated three times under the same conditions.

Based on bioassay-guided monitoring, the ethyl acetate extract of strain SCG was fractionated, and white active compound was obtained. The molecular weight and formula of active compound was determined as C_5_H_6_N_2_O_2_ by ESI-MS ([M + H]^+^, m/z 126.8) and 1D NMR spectra (1H and 13C NMR) (Fig. S2 and Table S2). The structure of active compound was readily identified as thymine by comparison with previously reported structures [35, 36]. Thymine isolated from strain SCG showed nematicidal activity in a concentration-dependent manner against *M. incognita* J2s, with mortalities 71.57% at a concentration of 0.5 mg/ml (Fig. 4).

### Effect of Strain SCG on *M. incognita* under Greenhouse Conditions

**Fig. 5:**
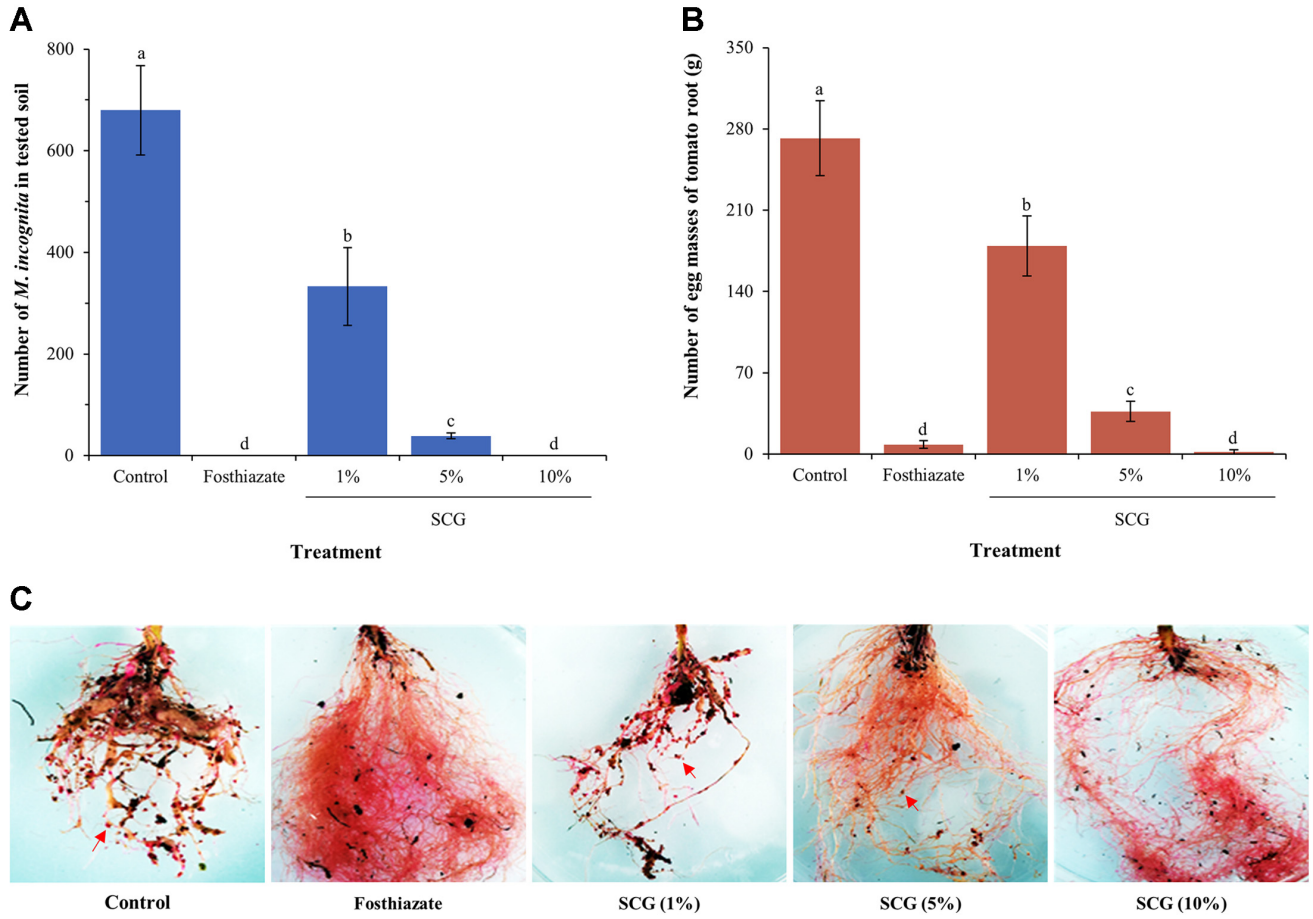
Effects of cell-free filtrates of SCG strain on the number of nematode populations (A) and egg masses (B) per plant in the pot experiment (*n* = 5). (C) Root symptoms of *S. lycopersicum*. The number of egg masses was determined using phloxine B staining. The red arrows indicate egg masses formed by *M. incognita* infection. The experiment was performed in triplicate under the same conditions. Different letters above the error bars indicate significant differences according to Scheffé's test (*p* < 0.05).

The population of *M. incognita* in the tested soil was observed after 45 days of treatment (Fig. 5A). The population of nematode in soil treated with 10% cell-free filtrates of SCG strain was significantly reduced (*p* < 0.005) compared with the control, and there was no statistically significant difference between SCG and the positive control, fosthiazate treatment (*p* > 0.05) (Fig. 5A). All tested concentrations reduced nematode populations as concentrations increased, demonstrating that these cell-free filtrates preserved their antagonistic effects in a concentration-dependent manner under greenhouse conditions. Similarly, the number of egg masses on each tomato root significantly decreased in a concentration-dependent manner compared to the control when treated with cell-free SCG filtrates (Fig. 5B and 5C). The 10% cell-free filtrates of strain SCG remarkably reduced the number of egg masses (2.0 ± 1.9 egg masses) similar with that of the positive control; in addition, fosthiazate (8.3 ± 3.4 egg masses) and other concentration decreased the number of egg masses in a concentration-dependent manner.

**Fig. 6:**
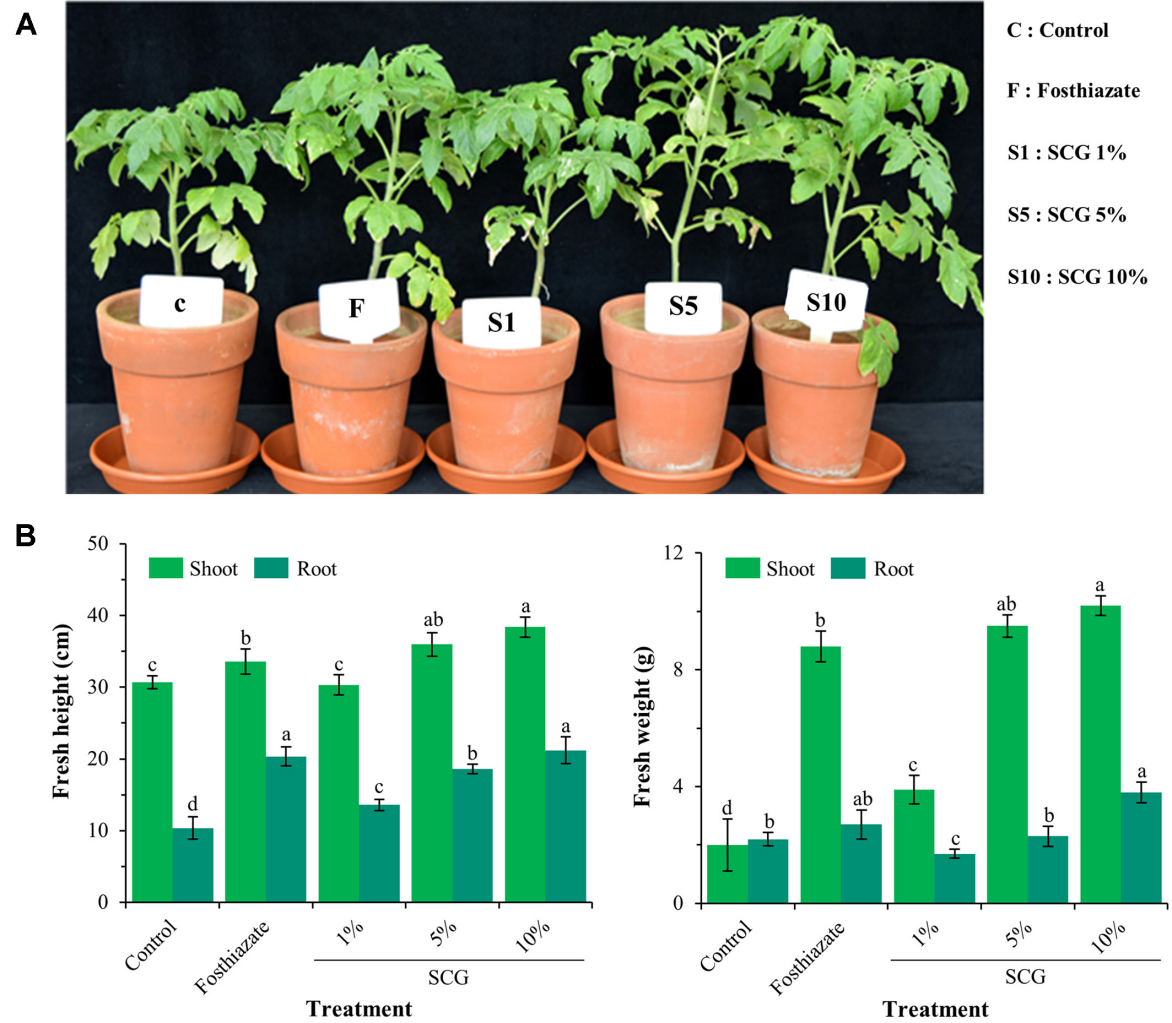
Effects of SCG strain cell-free filtrates on the growth of *S. lycopersicum* in the pot experiment (A). Fresh height and weight of the test *S. lycopersicum* was measured after 45 days of transplanting (**B**) (*n* = 5). The experiment was performed in triplicate under the same conditions. Different letters above the error bars indicate significant differences based on Scheffé's test (*p* < 0.05).

The application of 10% cell-free filtrates of strain SCG significantly affected all the plant growth parameters (fresh height and weight of shoots and roots) after 45 days and had a relatively optimized effect on tomato growth compared to positive control (fosthiazate) (Fig. 6). It yielded the highest fresh shoot length (38.4 ± 1.4 cm), fresh root length (21.2 ± 1.9), fresh shoot weight (10.2 ± 0.3), and fresh root weight (3.8 ± 0.4). However, the fresh root length did not differ significantly between 10% cell-free filtrates of SCG and fosthiazate (*p* > 0.05). Similar to the nematode parameters, the cell-free filtrates of strain SCG exhibited a marked dose-dependent effect on plant growth parameters.

## Discussion

Over the last several decades, eco-friendly nematicidal agents have been extensively studied to overcome the side effects of synthetic nematicides, such as toxic environmental hazards [37]. Recent studies suggest that the symbiotic bacterium of EPNs, *Xenorhabdus*, are one of the most untapped resources for biological nematicides to control PPNs [21, 38]. These bacteria have a mutualistic relationship with the entomopathogenic nematodes of the genus *Steinernema*. Following *Steinernema* invasion of the insect host hemocoel, they are released from the digestive tract of IJs and exhibit virulence by producing toxins and natural products, leading to host death [39]. Our results are consistent with those of a previous study, where seven *Steinernema* species isolated from forest soil samples exhibited high levels of insecticidal activities against 3rd instar larvae of *G. mellonella*. In the case of RKNs, recent studies reported that the nematicidal efficacy of the EPNs can be attributed to the presence of the symbiotic bacterium [40, 41]. Notably, bacterial species of the *Xenorhabus* genus can produce a wide variety of natural products with antimicrobial, insecticidal, and antiparasitic properties [42, 43]. This may indicate that *Xenorhapdus* species may play a crucial role in the virulence in EPNs-*Xenorhabdus* complexes [44]. These results show that *Xenorhabdus* species are potential sources of novel nematicidal agents. However, few studies have investigated the nematicidal activity of *Xenorhabdus* species against RKNs. In the present study, *Xenorhabdus* sp. SCG, which have excellent nematicidal activ-ity against the J2s of *Meloidogyne* species, were isolated and identified. The 10% cell-free filtrates of strain caused mortalities of > 80% on J2s of all four *Meloidogyne* species after 48 h exposure and exhibited significant nematicidal activities even against the three other genera of PPNs in a dose-dependent manner. Our findings are consistent with those reported by Abebew *et al*. who found that cell-free culture supernatants of Xenorhabuds bacteria showed high mortality rates for *Caenorhabditis elegans* and *M. javanica* [23]. In addition, the NPs, fabclavines, rhabdopeptides, and xenocoumacins from *Xenorhapdus* were highly toxic to *C. elegans* with mortalities of 95.3, 74.6, and 72.6%, respectively, and to *M. javanica* with mortalities of 82.0, 90.0, and 85.3%, respectively. Moreover, rhadopeptides from *Xenorhabdus budapestensis* SN84 exhibited strong inhibitory activity with LC_50_ values of 27.8 μg/ml (rhabdopeptide J), 46.3 μg/ml (rhabdopeptide K), and 42.4 μg/ml (rhabdopeptide M) [24]. The nematicidal activities of *Xenorhabdus* species have been studied; however, the present study is the first to demonstrate the nematicidal activity spectrum of isolated *Xenorhabdus* species and their application to *S. lycopersicum* L., cv. Rutgers under greenhouse conditions. In the present study, the nematicidal compound was purified by using bioassay-guided fractionation and thymine was identified. In accordance with our results, thymine isolated from the *Bacillus velezensis* RB.EK7 was reported to exhibit nematicidal activity [45].

Despite numerous advances in the laboratory studies of bacterial nematicides, extensive research is needed for their successful application under field conditions because of their lack of field adaptability and activity. Therefore, the discovery of new nematicidal strains from rhizospheric environments with high field activity and adaptability is essential for the sustainable control of RKNs. The results of the pot experiment indicated that the cell-free filtrates of the SCG strain could control *M. incognita* in a manner similar to that of chemical nematicides (fosthiazate), with plant growth-promoting effects in a dose-dependent manner under greenhouse conditions. These results suggest that NPs secreted by the SCG strain may have direct nematicidal effects or change soil ecosystems. Caccia *et al*. reported that *M. hapla*-infected tomato cv. Platelets treated with cell-free supernatants of *Xenorhabdus* sp. LB and *X. szentirmaii* RACA showed significantly reduced galls, egg masses, and nematode populations compared with the control group [25]. Similarly, the treatment of pecans (*Carya illinoensis*) with *X. bovienii* resulted in reduced galls, egg masses, and nematode populations, as well as increased dry root weight, suggesting its potential as a control agent for the pecan root-knot nematode *M. partityla* [46]. Considering all the results discussed above, this suggests that the strain SCG maintains its effect on soil conditions and may be used as a multi-functional agent. However, the strain needs to be further explored to determine the NPs. Moreover, additional field experiments are needed to allow for the proposal of more economical and ecologically favorable agents for sustainable agriculture.

Our study showed that *Xenorhabdus* sp. SCG, isolated from the entomopathogenic nematodes of the genus *Steinernema*, is a potential *M. incognita* control agent. Cell-free filtrates of the SCG strain showed high levels of nematicidal activity, with a broad spectrum of activity against PPNs, in a dose-dependent manner under in vitro conditions. Thymine isolated from strain SCG showed possible nematicidal activity in a concentration-dependent manner against *M. incognita*. In addition, cell-free filtrates of the SCG strain efficiently controlled *M. incognita* in infected tomato plants with plant growth-promoting effects under greenhouse conditions. These findings suggest that *Xenorhabdus* species could be a potential resource for novel nematicidal candidates. However, the major factors affecting the plant growth were limited in this study. Further research is required for the identification and verification of NPs from the SCG strain, as well as for the practical application of the strain in various field experiments. The results of this study elucidate the nematicidal potential of entomopathogenic bacteria in the management of RKNs and provide a theoretical basis for further studies.

## Supplemental Materials

Supplementary data for this paper are available on-line only at http://jmb.or.kr.
